# Whole-body phase angle correlates with pre-operative markers in total joint arthroplasty

**DOI:** 10.2478/joeb-2023-0008

**Published:** 2023-12-31

**Authors:** Michael C. Marinier, Ayobami S. Ogunsola, Jacob M. Elkins

**Affiliations:** 1 Department of Orthopedics and Rehabilitation, University of Iowa, Iowa City, IA USA

**Keywords:** Bioimpedance, whole body phase angle, arthroplasty, edema

## Abstract

**Background:**

Bioimpedance derived whole body phase angle (ϕ), a measure of cellular integrity, has been identified as an independent marker of morbidity and mortality in many medical and surgical specialties. While similar measures of water homeostasis like extracellular edema (EE) have been associated with pre-operative risk, ϕ has not been studied in orthopaedics, despite potential to serve as a pre-operative marker. This study aims to identify relationships between ϕ, EE, and body composition metrics, laboratory values, patient reported outcomes, and comorbidities.

**Methods:**

Multi-frequency bioimpedance analysis (BIA) records, laboratory values, and patient reported outcomes of adult patients presenting to an academic arthroplasty clinic were retrospectively reviewed. Correlation coefficients between ϕ, EE, and reviewed information were conducted.

**Results:**

ϕ was significantly correlated (p<0.001) most positively with measures of lean tissue such as skeletal muscle mass (r=0.48), appendicular skeletal muscle index (r=0.39), lean body mass (r=0.43), and dry lean mass (r=0.47), while it held negative correlations (p<0.001) with age (r= -0.55), and body fat mass (r= -0.11). ϕ was not correlated with body mass index (BMI, p = 0.204). In contrast, EE demonstrated its strongest positive correlations (p<0.001) with body fat mass (r=0.32), age (r=0.50), and BMI (r=0.26), and its strongest negative correlations (p<0.001) with serum albumin (r= -0.37) and total protein (r= -0.23).

**Conclusions:**

Based on their associations with markers of health and fitness, BIA determined ϕ and EE demonstrate relationships to markers currently implemented in orthopaedic practice. This likely indicates that ϕ has potential as a comprehensive surrogate for several commonly used markers to quantify pre-operative risk. In the future, ϕ may aid in developing risk-stratifications for intervention and prevention of complications.

## Introduction

Body size and habitus remain stalwarts in orthopaedics, and namely arthroplasty, as these patient metrics have long served as guides for surgical intervention [1–3]. However, assessment with conventional tools like body mass index (BMI, in kg/m^2^) is limited by its simplicity, which have contributed to concerns of its efficacy [4–6]. Efforts have been made to find alternative surrogates including muscle mass and body fat analyses, specifically overall fat mass and fat deposition characteristics, for predicting common post-operative complications [7–13]. These measurements can be determined from various modalities including dual x-ray absorptiometry (DEXA), CT scan, full-body MRI, and bioimpedance analysis (BIA) [11, 14–16].

Among these technologies, BIA additionally allows for complex body water analyses to be completed rapidly in a clinical setting [17–20]. BIA utilizes single or multiple frequency electrical impulses to obtain various tissues’ resistance (R) and reactance (X), which provides markers of intra- and extracellular water balance [18, 19, 21, 22]. Specifically, BIA produces whole-body phase angle (ϕ), which is marker of cellular membrane integrity via the function arctan(X/R), which has implications regarding cellular health. [23]

Previous reports have investigated ϕ as a surrogate to predict patient morbidity and mortality [24–26]. Largely within critical care, surgical oncology, cardiothoracic surgery, and geriatric medicine, decreasing ϕ has shown correlations with patient demise and adverse events. [24–29]. While it has been investigated in various surgical specialties, the utility of ϕ as an indicator of post-operative complications within the fields of orthopaedic surgery and musculoskeletal health has not been well described. Existing studies have primarily focused on geriatric native hip fractures: ϕ has been shown to predict the occurrence of hip fractures and post-fixation outcomes [30–32]. In arthroplasty, ϕ has not been previously investigated despite sharing desired outcomes and complications with the trauma population. Particularly, lymphedema, a condition that likely has pathophysiology linked to poor cellular integrity, has been attributed as a risk factor for poor outcomes following arthroplasty [33]. This paucity of information surrounding ϕ in arthroplasty provides ample open avenues for studies.

## Materials and methods

This study is a retrospective review of adult patients (>18 years) who presented to arthroplasty clinic at an academic hospital and underwent multi-frequency BIA (InBody 770; InBody USA; Cerritos, CA USA) from 1/1/2020 to 3/31/2022. The InBody 770 employs six frequencies for impedance measurements (1, 5, 50, 250, 500, 1000 kHz) and three frequencies for reactance measurements (5, 50, 250 kHz). To complete the BIA exam, a tetrapolar eight-point electrode configuration is employed: patients stand with the plantar aspect of each foot contacting two electrodes, and each hand also contacts two electrodes (one at the thumb and one for remaining digits).

Patients routinely undergo BIA upon their first presentation to clinic with exception if they are unable to stand for approximately 60 seconds and/or have an implanted electronic cardiac device; however, it is unclear if presence of an implanted electronic cardiac device is a true contraindication to BIA [34, 35].

Patients’ BIA data including ϕ, extracellular edema (EE), skeletal muscle mass (SMM), body fat mass (BFM), lean body mass (LBM), dry lean mass (DLM), appendicular skeletal muscle index (aSMI), and visceral fat area (VFA) were reviewed for analysis. Additionally, subjects’ recent laboratory values, and patient reported outcomes (PROs) were collected. Laboratory value within five weeks of the BIA scan were recorded, including hemoglobin (Hgb), serum albumin, and serum total protein.

Statistical analyses were completed using SAS (SAS Institute; Cary, NC USA) and Microsoft Excel (Microsoft Corporation; Redmond, WA USA). The primary analysis included calculation of correlation coefficients between the above variables and ϕ. The secondary analysis included calculation of correlation coefficients between the above variables and EE. Statistical significance was delineated at p < 0.05.

### Ethical approval

The research related to human use has been complied with all relevant national regulations, institutional policies and in accordance with the tenets of the Helsinki Declaration and has been approved by the authors’ institutional review board or equivalent committee.

## Results

Six hundred ninety-five patients who underwent BIA were identified for analysis including 332 males (47.8%) and 363 females (52.2%). Mean age was 61.00 ± 12.94 years. Patient demographics and pre-operative BIA measurements are included in Table 1.

**Table 1:** Demographics.

**Demography of Study Participants N = 695**	**N or Mean (SD)**	**Percent**
**Gender**		
Males	332	47.8
Females	363	52.2
Age, Yr	61 (12.94)	-
BMI, kg/m^2^	33.93 (8.3)	-
**Bioimpedance Analysis Measurements**	**N or Mean (SD)**	**Percent (SD)**
**Skeletal Muscle Mass, kg**		
Male	38.61 (6.60)	-
Female	26.74 (5.01)	-
**Appendicular Skeletal Muscle Index, kg/m^2^**		
Male	9.38 (1.17)	-
Female	7.55 (1.32)	-
Phase Angle, Degrees	4.82 (0.88)	-
Extracellular Edema, %	-	39.4 (1.2)
Dry Lean Mass, kg	38.61 (3.63)	-
Lean Body Mass, kg	58.71 (14.06)	-
Visceral Fat Area, cm^2^	184.24 (73.64)	-
Body Fat Mass (±SD), g	39.21 (18.67)	-
**Laboratory Measurements**	**N or Mean (SD)**	**Percent**
Hemoglobin (g/dl)	13.3 (1.73)	-
Serum Total Protein (g/dl)	7.04 (0.54)	-
Serum Albumin (g/dl)	4.30 (0.41)	-
Serum Creatinine (mg/dl)	0.94 (0.36)	-
Serum Calcium (mg/dl)	9.21 (0.53)	-
Blood Urea Nitrogen (mg/dl)	17.19 (8.21)	-
**Patient Reported Outcomes**	**N or Mean (SD)**	**Percent**
Mental Health Score	43.19 (14.67)	-
Physical Health Score	37.50 (12.22)	-
General Health Score	3.07 (0.83)	-

**Figure 1:**
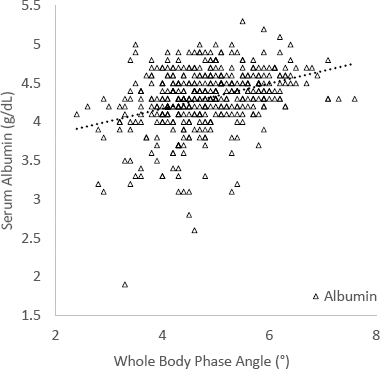
Scatter plot demonstrating serum albumin relative to ϕ. Serum albumin, a common marker of nutrition utilized in joint arthroplasty, correlated with significantly with ϕ (*r* = 0.34, *p* < 0.001).

**Figure 2:**
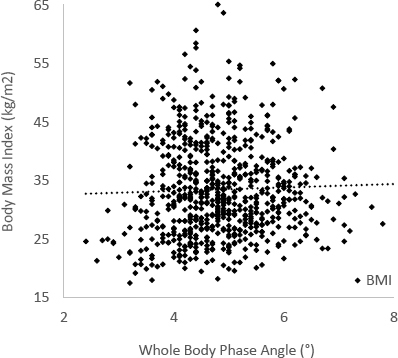
Scatter plot demonstrating BMI relative to ϕ. BMI has long been used as a pre-operative predictor of post-arthroplasty complications, but it did not demonstrate a significant correlation with ϕ (*r* = 0.05, *p* = 0.204).

In the entire sample, the mean ϕ was 4.82 ± 0.88°. The largest positive correlation coefficients with ϕ include SMM (r = 0.48, p < 0.001), DLM (r = 0.47, p < 0.001), and LBM (r = 0.43, p < 0.001). Serum albumin was the most positive correlation coefficient at r = 0.43 (p < 0.001), among the selected laboratory values (Fig. 1). EE (r = -0.79, p < 0.001), age (r = -0.55, p < 0.001), and percent body fat (PBF, r = -0.31, p < 0.001) were the most negative correlation coefficients identified. All correlation coefficients with ϕ are summarized in Table 2. BMI (r = 0.05, p = 0.204) was not identified as a significant correlator with ϕ (Fig. 2). Among the PROs, only general health score demonstrated a significant, yet weak, relationship (r = 0.12, p = 0.01), while mental and physical health scores did not.

**Table 2:** Pearson Correlation between Phase Angle (left), Extracellular Edema (right), and other study participants characteristics.

	Phase Angle		Extracellular Edema	
Variable	Correlation Coefficients	P-value	Correlation Coefficients	P-value
Age	-0.55	<.0001*	0.50	<.0001*
BMI	0.05	0.204	0.26	<.0001*
Extracellular Edema	-0.79	<.0001*	-	-
Skeletal Muscle Mass	0.48	<.0001*	-0.08	0.029*
Dry Lean Mass	0.47	<.0001*	-0.08	0.044*
Lean Body Mass	0.43	<.0001*	-0.02	0.653
Visceral Fat Area	-0.16	<.0001*	0.26	<.0001*
Skeletal Muscle Index**	0.39	<.0001*	0.14	<.001*
Percent Body Fat	-0.31	<.0001*	0.31	<.0001*
Body Fat Mass	-0.11	<.001*	0.32	<.0001*
Physical Health Score	-0.09	0.09	-0.12	0.021*
Mental Health Score	-0.02	0.71	-0.00	0.941
General Health Score	0.12	0.01*	-0.20	<.0001*
Serum Calcium	-0.01	0.76	-0.06	0.185
Serum Creatinine	0.03	0.69	0.07	0.149
Serum Total Protein	0.26	<.0001*	-0.23	<.0001*
Serum Albumin	0.34	<.0001*	-0.37	<.0001*
Blood Hemoglobin	0.33	<.0001*	0.15	0.04*

* *Significant correlations are less than 0.05*.

** *Appenidicular Skeletal Muscle Index*

1 *Correlation values of ≥ 0.75 are strong and values ≤ 0.5 are weak (values in between are moderate)*.

Whole-body EE was determined to be 0.394 ± 0.012, on average. Positive correlators with EE include age (r = 0.50, p < 0.001), BFM (Fig. 3, r = 0.32, p < 0.001), and VFA (r = 0.26, p < 0.001). Serum albumin (r = -0.37, p < 0.001) and serum total protein (r = -0.23, p < 0.001) held the most negative correlation coefficients with EE (Table 2). SMM (r = -0.08, p = 0.029) and DLM (r = -0.08, p = 0.044) were significantly but very weakly correlated with EE. In contrast to ϕ, EE demonstrated a more significant correlation with the PROs and namely the general health score (r = -0.20, p < 0.001) and physical health score (r = -0.12, p = 0.021).

## Discussion

This study aimed to investigate potential relationships between BIA-determined markers of water balance such as ϕ and EE and common clinical and body composition metrics. While ϕ has been shown to have correlations with increased levels of inpatient morbidity and mortality, such relationships have not been investigated in orthopedics or the realm of elective surgery. Similarly, patients with edematous extremities have been recently identified as markedly more at risk for complications following arthroplasty procedures [18, 33, 36].

While conventionally identified purely through physical exam, EE can be objectively quantified using BIA with potential to be used as a guide in offering operative intervention and providing counseling regarding patient-specific risks for peri-operative complications.

As a marker of cellular integrity, ϕ was highly and significantly correlated with markers of lean tissue including SMM, DLM, and LBM. Among SMM and aSMI, female patients exhibited stronger correlations than males, which may indicate that female patients are more susceptible to the adverse effects of sarcopenia, which is a deficit of muscle mass or function [37]. In contrast to the lean tissue markers, the traditional risk stratification tool in arthroplasty, BMI, did not exhibit a significant relationship with ϕ. The absence of a correlation between BMI and ϕ may corroborate recent reports that question the increased incidence of post-operative complications in patients with elevated BMI [1, 2, 7, 38]. Additionally, fat surplus denoted by BFM and PBF were weaker correlators with ϕ compared to the lean tissue masses. These findings imply that conventional assessments focusing purely on body size may not be as predictive as patients’ unique body composition. Serum albumin and total protein have also long been nutritional surrogates to identify potential at-risk patients, and these measurements did significantly and positively correlate with ϕ. In addition, blood hemoglobin showed a similar relationship, which may indicate that increased aerobic fitness predisposes surgical patients to improved outcomes [39–41]. Finally, this study corroborates previous reports that increasing age is associated with decreasing ϕ [25, 33, 42, 43].

In contrast to the relationships identified with ϕ, EE demonstrated its strongest correlations with markers of fat mass rather than lean mass. These fat markers included overall BFM and VFA, where the latter may provide an objective marker of metabolic syndrome and lipedema, which are known as additional risk factors for complications. Similarly, low nutritional serum markers correlated with increased edema. In addition to being surrogates for nutritional status, serum albumin and total protein affect fluid homeostasis through oncotic pressure, so age was also identified as a positive correlator with EE. In general, EE can be signs of tissue inflammation and sedentary lifestyles, which are characteristics that likely contribute to these findings.

This study is not without limitations. First, this study is a retrospective review of BIA data collected at a single academic center. Furthermore, there were not set enrollment criteria, and the population was largely affected by osteoarthritis or similar hip or knee pathology. Finally, laboratory values and PROs were not uniformly collected at the time of BIA.

Future directions include longitudinal risk assessment of patients undergoing TJA using ϕ and EE as pre-operative markers and studies that identify at risk patients and provide nutritional and exercise interventions. Many of these studies are currently underway.

In conclusion, measures of impaired water balance, decreasing ϕ and increasing EE, demonstrate numerous correlations with markers of physical health decrement such as decreasing lean mass and increasing fat mass. These measurements are sparingly utilized in arthroplasty currently but have been identified as risk markers. ϕ and EE via BIA provides promising objective pre-operative assessment that may be a comprehensive surrogate for existing risk factors.
